# Direct and indirect effect of 10 valent pneumococcal vaccine on nasopharyngeal carriage in children under 2 years of age in Matiari, Pakistan

**DOI:** 10.1016/j.vaccine.2020.12.066

**Published:** 2021-02-22

**Authors:** Muhammad Imran Nisar, Sheraz Ahmed, Fyezah Jehan, Shahira Shahid, Sadia Shakoor, Furqan Kabir, Aneeta Hotwani, Sahrish Munir, Sajid Muhammad, Farah Khalid, Benjamin Althouse, Hao Hu, Cynthia Whitney, Asad Ali, Anita K.M. Zaidi, Saad B. Omer, Najeeha Iqbal

**Affiliations:** a Department of Pediatric and Child Health, Aga Khan University, Karachi, Pakistan; b Department of Pathology and Laboratory Medicine, Aga Khan University, Karachi, Pakistan; c Bill & Melinda Gates Foundation, Seattle, WA, USA; d Emory University, Atlanta, GA, USA; e Yale Institute for Global Health, New Haven, CT, USA

**Keywords:** Streptococcus pneumoniae, Pneumococcal vaccines, Herd immunity, Pakistan

## Abstract

**Background:**

Pakistan introduced Ten-valent pneumococcal-conjugate-vaccine PCV10 in 2012 as a 3 + 0 schedule without catch-up.

**Methods:**

Children <2 years old in Matiari, Sindh provided nasopharyngeal swabs between 2014 and 2018, which were cultured for pneumococcus and serotyped through multiplex PCR at the Aga Khan University Hospital. Carriage rates over time for Vaccine-Type (VT) and Non-VT (NVT) serotypes were used to estimate direct, indirect, total and overall effects of vaccination. Regression analysis was used to determine factors associated with VT carriage.

**Results:**

Pneumococcus was detected in 2370/3140 (75%). VT carriage decreased overall, 16.1–9.6% (p-trend <0.001); vaccinated (all 3 doses of PCV10 received) 11.3–8.1% (p-trend 0.031) and unvaccinated (no PCV10 dose received) 17.4–10.3% (p-trend 0.003) with a decline in serotypes 6B, 9V/9A and 19F. Immunization increased from 41.0% to 68.4% (p-trend 0.001). Direct effect of vaccine was 32.8% (95% CI 14.7–47.0%) and indirect effect 44.6%(95% CI 40.6–48.6%). Factors associated with decreased VT colonization were education 1–5 years (aOR 0.7, 95%CI 0.6–1.0), history of difficulty breathing (aOR 0.7, 95%CI 0.5–1.0), exposure to smoke (aOR 0.8, 95% CI 0.6–1.0), child fully immunized (aOR 0.7, 95%CI 0.5–1.0) and enrolled in 3rd (aOR 0.6, 95%CI 0.4–0.8) and 4th (aOR 0.6, 95%CI 0.5–0.9) year of the study whereas history of runny nose (aOR 1.5, 95% CI 1.2–1.9) was positively associated.

**Conclusions:**

Decrease in VT pneumococcal carriage in vaccinated and unvaccinated children indicates herd immunity. Sustained increase in vaccine coverage and close long-term surveillance is warranted.

## 1 Introduction

Pneumonia, caused by a number of infectious agents, is the leading cause of morbidity and mortality in children worldwide. *Streptococcus pneumoniae* (pneumococcus) is the leading bacterial cause contributing to more deaths than all other etiologies combined [1]. There were an estimated 138 million (86–226 million) episodes of clinical pneumonia and 0.9 million (0.8–1.1 million) deaths in children less than five years of age in the year 2015 globally [2]. Although the incidence and mortality from pneumonia has fallen globally during the Millennium Development Goal (MDG) era (2000–2015), in Pakistan the incidence has increased by 50%. There were around 7.1 million (4.2–12.0 million) episodes of clinical pneumonia and around 63,960 pneumonia deaths in children under the age of five years in Pakistan in the year 2015 [2]. Deaths due to pneumococcal infections were 318,000 (207,000–395,000) in this age group with 14,000 (9700–17,000) of these occurring in Pakistan [3].

Pneumococcus has more than 90 known serotypes and resides asymptomatically in the upper respiratory tract of humans [1], [4], [5]. Colonization may be present as early as first few months of life [6]. In some cases, colonization progresses to invasive or non-invasive disease with most caused by a limited number of serotypes [7]. The most common of these serotypes responsible for causing invasive disease are included in pneumococcal conjugate vaccines like PCV10 and PCV13 [7]. As of June 2020, 146 countries, including 60 with support from Gavi, the Vaccine Alliance, have introduced pneumococcal conjugate vaccine in their countries’ immunization program [8]. Various post introduction impact evaluation studies have subsequently shown significant decline in invasive disease as well as carriage. Benefit in many cases have spilled over to adult population as well as older children and children too young to be vaccinated [9], [10], [11]. In 2012, Pakistan became the first country in South Asia to introduce ten valent pneumococcal vaccine (PCV10) in its Expanded Program on Immunization (EPI) with WHO recommended schedule of three doses given at 6, 10 and 14 weeks of life (3 + 0 schedule). No catch-up immunization was offered [12], [13].

In this paper, we aim to describe impact of PCV10 on pneumococcal carriage in vaccinated (direct protection) and unvaccinated children (indirect or herd protection) aged less than two years in a rural population in Matiari, Sindh, Pakistan. We use point estimate of 26.7% as the baseline Vaccine Type (VT) carriage from a pre-introduction carriage survey in the same population [14]. We also describe the socio-demographic and clinical characteristics associated with VT carriage.

## 2 Methods

### 2.1 Study design and participants

This study was carried out from October 2014 to September 2018 in two Union Councils (Khyber and Shah Alam Shah Jee Wasi) of Matiari in Sindh, Pakistan, having a total population of around 88,739. Matiari is a rural district located 180 km from the Aga Khan University in Karachi. Average household size is 7, literacy rates are low with most of the population engaged in agriculture. The site was chosen because we had previously done a pre-PCV10 introduction carriage survey in the same population in 2012–13, which provided an estimate of vaccine type pneumococcal carriage in a vaccine naïve population. PCV10 was introduced in this population in early part of 2013 as per the EPI schedule with no catchup immunization.

A background demographic surveillance system (DSS) in the area provided a frame-work for randomly selecting 15 age eligible children every week from the available line listing. Each child was only enrolled once in the study. There were no significant supply chain issues related to PCV10 encountered during the course of the study. Vaccination history of the child was collected as a combination of caregiver reported or/and card verified (where available). PCV10 and pentavalent vaccine follow the same schedule in Pakistan’s EPI and it is a common practice for the vaccinators to give PCV10 in the left and pentavalent in right thigh. In instances where children received only one of the two recommended shots, the caregivers were enquired about the site of the injection.

Children with nose and throat abnormalities or with a serious illness requiring hospitalization were excluded from the study. Data on household demographics, recent clinical history including hospitalization and outpatient visits, exposure to household smoke and indoor air pollution was collected by study personnel on smartphones. A brief clinical exam including measurement of fever, respiratory rate and observation for chest wall indrawing was also done.

### 2.2 Nasopharyngeal swab collection

Nasopharyngeal specimens were collected and transported at 2–8 °C from the field site to the Infectious Disease Research Laboratory (IDRL) in Karachi within 8 h of collection using established World Health Organization’s (WHO) consensus methods [15].

### 2.3 Laboratory procedures

In the lab, samples were vortexed for 10–20 s to disperse the organism and afterwards frozen at −80 °C in an upright position till time of further processing. For culture (batches of 20–40), samples were thawed, vortexed and 200 µL of a sample was added to mixture of 1 mL rabbit serum, 5 mL Todd Hewitt broth with 0.5% yeast extract and incubated for 6 h at 37 °C. After this, one loop full (10 ul) was inoculated onto bilayer sheep blood and colistin-nalidixic-acid-agar and streaked for isolation of streptococci. After 18–24 h, plates were examined for the appearance of alpha-hemolytic colonies and susceptibility to optochin and bile solubility. Serotypes were deduced using the published sequential multiplex PCR assay and further confirmation was done by monoplex PCR [16], [17]. Resolution of sero-group 6A/B/C/D was done as per the methods described by Ping et al [18]. Identification of non-typeable pneumococcal isolates (those with no positive results for any of the 39 serogroups/ types) were confirmed with a *lytA* Real time PCR as described by Carvalho MDG et al. [19]. Further description of the laboratory methods is given in Figure s6-s8 in the supplementary material.

### 2.4 Statistical analysis

Vaccine Type (VT) carriage was defined as isolation of any of the 10 serotypes included in PCV10 (serotypes 1, 4, 5, 6B, 7F, 9V, 14, 18C, 19F, 23F). Non-vaccine type (NVT) carriage was defined as presence of all other pneumococci including the non-typeables. PCV13 serotypes were defined as any of the PCV10 serotypes plus serotypes 3, 6A and 19A. A study year ran from October to the September of next year. We describe carriage rates by number of the doses of the vaccine received and study year.

**Fig. 1:**
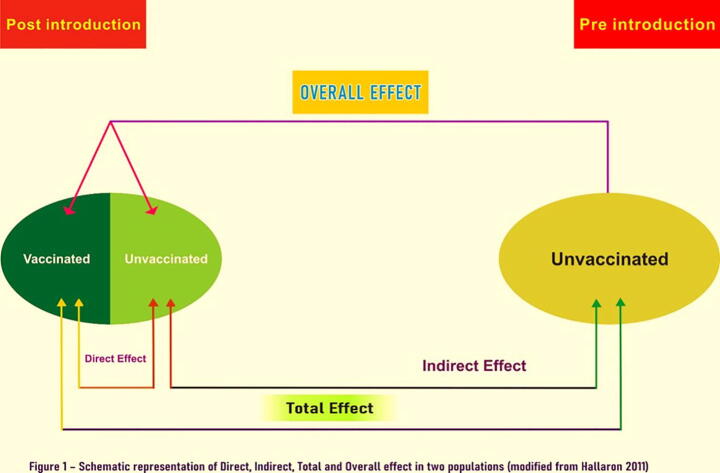
Schematic representation of direct, indirect, total and overall effect in two populations (modified from Halloran 2011) Direct effect was calculated as 1– (VT carriage rate in children who received all three doses /VT carriage rate in children who received zero dose), indirect effect was defined as 1- (VT carriage rate in children who received zero dose /26.7%), total effect was defined as 1-(VT carriage rate in children who received all three doses/26.7%) and overall effect was defined as 1- (carriage rate in the study population / 26.7%).

Measures of direct, indirect, total, and overall effects were calculated using a modified Halloran Model described in Fig. 1. For the estimate of carriage rate in a vaccine naïve population (the unvaccinated compartment in the Halloran model), we used VT carriage rate from a cross-sectional survey carried out in the same population in January- February 2013 prior to introduction of PCV 10. Following definitions for the population-level impact of PCV10 VT carriage were used: Direct effect was calculated as 1– (VT carriage rate in children who received all three doses/VT carriage rate in children who received zero dose); indirect effect was defined as 1- (VT carriage rate in children who received zero dose /26.7% which was the pre-introduction VT carriage rate), total effect was defined as 1-(VT carriage rate in children who received all three doses/26.7%) and overall effect was defined as 1- (carriage rate in the study population /26.7%). As a part of sensitivity analysis, we excluded children who were too young to be fully vaccinated i.e. <4 months of age and also compared VT carriage rate in those who received 1 or more doses of PCV10 against those who did not receive any dose. Logistic regression analysis was performed to identify predictors of colonization with a PCV10 serotype. An additional analysis was done to describe predictors of overall pneumococcal carriage in these children. For the purpose of model building all variables with a p-value less than 0.25 in the bivariate analysis were used to build a multivariable model. A backward selection procedure was used to derive a parsimonious model for retaining only variables significant at p-value ≤ 0.05. All analysis was performed using STATA version 15.0.

Ethical approval was obtained from Aga Khan University’s Ethical Review Committee (3181-Ped-ERC-14). A written informed consent was obtained from all caretakers before commencing enrollment.

### 2.5 Role of funding source

This study was funded by Bill & Melinda Gates Foundation through grant # OPP1111303. Funder had no role in collection, analysis or interpretation of the data.

## 3 Results

### 3.1 Characteristics of the study participants

**Fig. 2:**
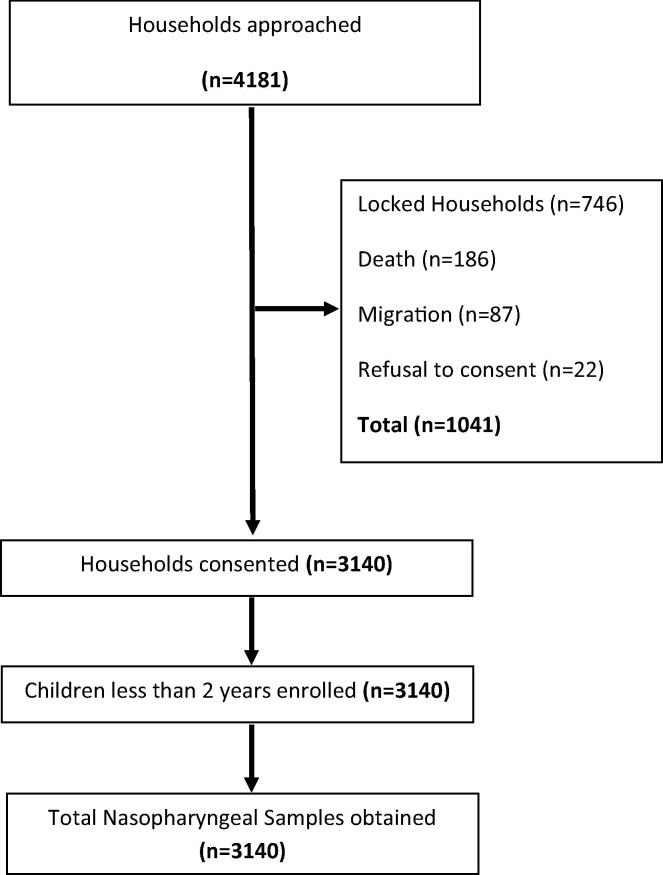
Flowchart describing study population.

We approached a total of 4181 households during the 4-year study period from which 3140 children under the age of 2 years, meeting our study criterion were enrolled after obtaining a written informed consent from the primary care giver. The flow of the children into the study is described in Fig. 2. Table 1 describes socio-demographic and clinical history of the enrolled children. Mean age was 10.5 months, half of the enrolled children were male, the number of children too young to be fully vaccinated (≤4months) were 304 (9.7%), majority of the primary caretakers 2596 (82.7)% and half of the primary wage earners, 1671 (53.2%) had no education at all; median household size was 8; one third of children were exposed to environmental tobacco smoke (ETS); natural gas was available in only 15% of the household and nearly half of the children were exposed to indoor air pollution by cooking. History of cough, runny nose and fever in past two weeks was common. Six percent had fever at the time of enrollment, 7% had tachypnea and 1.5% had chest wall indrawing. Vaccination cards were available for 2388 (76.05%) children.

**Table 1:** Socio-demographic and clinical characteristics of enrolled children, n = 3140.

Characteristics	n (%)
Age	
Mean (SD) in months	10.5 (6.0)
0–3 months	304 (9.7)
4–11 months	1604 (51.1)
12–23 months	1232 (39.2)
Males	1580 (50.3)
Primary caretaker education	
No education	2596 (82.7)
1–5 years	351 (11.2)
6–16 years	193 (6.1)
Primary wage earner education	
No education	1671 (53.2)
1–5 years	761 (24.2)
6–16 years	708 (22.5)
People in the household, median (IQR)	8 (6–11)
Number of rooms in house, median (IQR)	1 (1–2)
Crowding index *, median (IQR)	5.5 (4–7)
Hospital Admissions in last year	
None	3046 (97.0)
One	83 (2.6)
Two or more	11 (0.4)
Outpatient visits in last month	
None	1611 (51.3)
One	797 (25.4)
Two or more	732 (23.3)
Smoker in household	1116 (35.5)
Fuel used for cooking	
Wood/paper/straw/crop/animal dung	2640 (84.1)
Natural Gas	485 (15.4)
Other	15 (0.5)
Child exposed to smoke during cooking	1689 (53.8)
Symptoms during last two weeks**	
Runny nose	1 584 (51.6)
Cough	1 220 (39.8)
Fever	1 463 (47.7)
Fast breathing	79 (2.6)
Difficulty in breathing	612 (19.9)
Lower chest indrawing	74 (2.4)
Signs**	
Hypothermia <35 °C	11 (0.4)
Hyperthermia >37.5	187 (6.1)
Tachypnea***	210 (6.9)
Lower chest indrawing	47 (1.5)

IQR = Interquartile Range, * defined as no. of persons/room, **data available for 3068 children, ***using WHO age-specific cutoffs.

### 3.2 Carriage prevalence

**Fig. 3:**
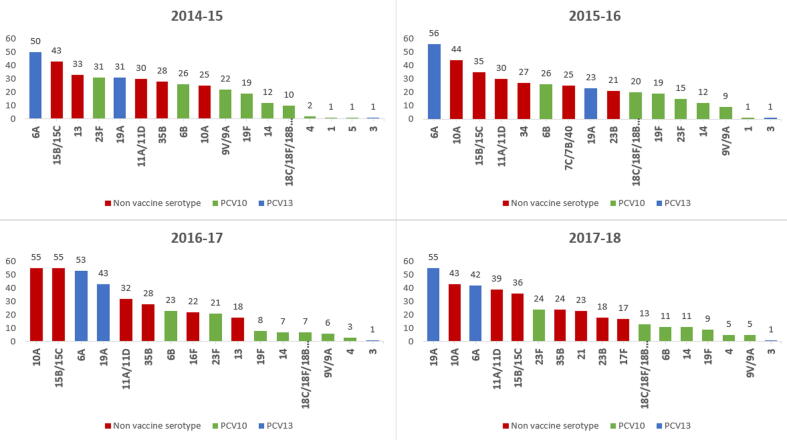
Ten most prevalent Vaccine-Type and NVT serotype distribution over the years (2014–2018).

All of the collected samples were analyzable. Pneumococcus was detected in 2370 children (75.5%; 95% CI 74.0–77.0). Out of these we were able to assign a serotype to 2155 (90.9%). Fig. 3 shows the VT and the ten most prevalent Non-Vaccine Type (NVT) serotypes over the study period. Pneumococcal carriage decreased from 80.8% in year 2014/15 to 72.8% in year 2017/18 (p-value for trend 0.001 - Table 2 and Figure s1). VT carriage decreased from 16.1% in year 2014/15 to 9.6% in 2017/18 (p-value for trend <0.001). There was no change in prevalence of the three serotypes included in PCV13 specific serotypes (3, 6A and 19A) or NVT serotypes (p-value for trend = 0.202 and 0.999 respectively). Concurrently the proportion of children vaccinated with all three doses of PCV10 (as per verbal report or card verification) increased from 41.0% to 68.4%. (Table 3). In children who received all 3 doses of PCV10, VT carriage decreased from 11.4% (95% CI 8.1–15.4) in 2014 to 8.1% (95% CI 6.0–10.7) in 2018 (p-value for trend 0.031) while in children who received one and two doses VT carriage decreased from 24.7% (95% CI 16.0–35.3) to 16.4% (95% CI 8.8–27.0) and from 20.0% (95% CI 12.7–29.2) to 12.3% (95% CI 6.7–20.1) respectively (p-value for trend 0.068 and 0.172) and in children who never received a PCV10 dose, VT decreased from 17.4% (95% CI 13.1–22.5) in 2014 to 10.4%(95% CI 4.6–19.4) in 2018 (p-value for trend 0.003) (Table 4). Table s3 and s5 in the supplement give VT carriage by number of doses and by age group.

**Table 2:** Vaccine Type and Non-Vaccine Type Carriage rate over time.

Year	2014/15 (n = 771)	2015/16 (n = 780)	2016/17 (n = 779)	2017/18 (n = 810)	2014–2018 (n = 3140)
No. positive for pneumococcus	623	574	583	590	2370
Prevalence of pneumococcus (95% CI)*	80.8 (77.8–83.5)	73.6 (70.3–76.7)	74.8 (71.6–77.9)	72.8 (69.6–75.9)	75.5 (74.0–77.0)
No. positive for PCV10 serotypes	124	102	75	78	379
Prevalence of PCV 10 serotypes (95% CI)^€^	16.1 (13.6–18.9)	13.1 (10.8–15.6)	9.6 (7.6–11.9)	9.6 (7.7–11.9)	12.1 (11.0–13.3)
No. positive for 3 PCV13 serotypes	82	80	97	98	357
Prevalence of additional PCV 13 serotypes (95% CI)^∞^	10.6 (8.5–13.0)	10.3 (8.2–12.6)	12.5 (10.2–15.0)	12.1 (9.9–14.5)	11.4 (10.3–12.5)
No. positive for NVT serotypes	499	473	508	510	1990
Prevalence of NVT serotypes (95% CI)^π^	64.7 (61.2–68.1)	60.6 (57.1–64.1)	65.2 (61.8–68.6)	63 (59.5–66.3)	63.4 (61.7–65.1)

*p value for trend is 0.001 **^€^**p value for trend is <0.001 **^∞^** p value for trend is 0.202 ^π^p value for trend is 0.999.

**Table 3:** Number of vaccine doses received among study participants by year.

No. of PCV doses	2014–2015 (n = 771)	2015–2016 (n = 780)	2016–2017 (n = 779)	2017–2018 (n = 810)	2014–2018 (n = 3140)
0 dose % (95%CI)	35.0 (31.7–38.5)	19.9 (17.1–22.8)	11.0 (8.9–13.5)	9.5 (7.6–11.7)	18.7 (17.4–20.1)
1 dose % (95%CI)	11.0 (8.9–13.4)	13.8 (11.5–16.5)	10.9 (8.8–13.3)	9.0 (7.1–11.2)	11.2 (10.1–12.3)
2 doses % (95%CI)	13.0 (10.7–15.5)	11.7 (9.5–14.1)	12.1 (9.9–14.6)	13.1 (10.8–15.6)	12.5 (11.3–13.7)
3 doses**% (95%CI)**	41.0 (37.5–44.6)	54.6 (51.0–58.2)	66.0 (62.5–69.3)	68.4 (65.1–71.6)	57.6 (55.9–59.4)

p-value for trend 0.001.

**Table 4:** Direct, Indirect, Total and Overall effect of PCV10 on VT carriage (0 dose vs 3 doses).

Year	2014–2015	2015–2016	2016–2017	2017–2018	2014–2018
VT carriage (3 doses) % (95% CI) *	11.4 (8.1–15.4)	12.4 (9.5–16.0)	8.9 (6.6–11.8)	8.1 (6.0–10.7)	9.9 (8.6–11.4)
VT carriage in 2 doses % (95% CI) ^β^	20.0 (12.7–29.2)	12.1 (6.2–20.6)	7.4 (3–14.7)	12.3 (6.7–20.1)	13.0 (9.9–16.8)
VT carriage in 1 dose % (95% CI) ^α^	24.7 (16.0–35.3)	14.8 (8.7–22.9)	14.1 (7.5–23.4)	16.4 (8.8–27.0)	17.4 (13.6–21.8)
VT carriage (0 doses) % (95% CI)^∞^	17.4 (13.1–22.5)	14.2 (9.1–20.7)	11.6 (5.7–20.3)	10.4 (4.6–19.4)	14.8 (12.0–17.9)
Direct Effect (95% CI)	34.6 (2.1–56.2)	12.3 (-39.1–44.8)	23.0 (-46.6–59.60)	21.8 (-59.5–61.68)	32.8 (14.7 – 47.0)
Indirect effect (95% CI)	34.8 (29.1–40.5)	46.8 (39.0–54.7)	56.4 (46.0–66.9)	61.1 (50.2–72.0)	44.6 (40.6–48.6)
Total effect (95% CI)	57.3 (51.9–62.8)	53.4 (48.7–58.1)	66.5 (62.4–70.6)	69.6 (65.7–73.4)	62.8 (60.5–65.0)
Overall effect (95% CI)	39.8 (36.3–43.2)	51.0 (47.5–54.5)	63.9 (60.6–67.2)	63.9 (60.6–67.2)	54.8 (53.1–56.5)

VT carriage prior to PCV10 introduction was taken as 26.7% *p-value for trend 0.031, ^α^ p-value for trend 0.172, ^β^ p-value for trend 0.068, ^∞^p-value for trend 0.003.

**Direct effect was calculated as 1– (VT carriage rate in children who received all three doses /VT carriage rate in children who received zero dose), indirect effect was defined as 1- (VT carriage rate in children who received zero dose /26.7%), total effect was defined as 1-(VT carriage rate in children who received all three doses/26.7%) and overall effect was defined as 1-** (**carriage rate in the study population / 26.7%).**

### 3.3 Direct and indirect effects

Table 4 describes various measures of vaccine effectiveness. In aggregate, the direct effect of the vaccine was calculated to be 32.8% (95% CI 14.7–47.0) and indirect effect was calculated to be 44.5% (95% CI 40.6–48.6). Total effect was shown to be 62.8% (95% CI 60.5–65.0%) and overall effect to be 54.8% (95% CI 53.1–56.5%). Table 4 also gives the variation in these effect estimates over the years. Table s4 give the sensitivity analysis by including children receiving ≥1 dose as vaccinated. This only slightly changed the estimates for direct and total effect. Table s5 and s6 describe the same analysis after dropping children who were too young to have received all three doses of PCV10 (<4 months of age). There were slight changes in the estimates which did not have a significant impact on the overall results.

### 3.4 Risk factors for VT carriage

Table 5 shows association of different variables with PCV 10 type carriage. In the final adjusted model, history of a runny nose in past two weeks (aOR 1.5, 95% CI 1.2–1.9) was positively associated with VT carriage. On the other hand, primary wage earner having 1–5 years of education (aOR 0.7, 95% CI 0.6–1.0), history of difficulty in breathing in past two weeks (aOR 0.7, 95%CI 0.5–1.0), exposure to environmental tobacco smoke (aOR 0.8, 95% CI 0.6–1.0), having received 3 doses of PCV10 (aOR 0.7, 95%CI 0.5–1.0) and being enrolled in 3rd (aOR 0.6, 95%CI 0.4–0.8) and 4th year (aOR 0.6, 95%CI 0.5–0.9) of the study were negatively associated with VT carriage.

**Table 5:** Predictors of PCV10 serotype carriage in children <2 years of age, n = 3140.

	PCV 10 negative		PCV 10 positive			Adjusted Model
	n(%)		n(%)		OR (95% CI)	aOR (95% CI)
N	2761		379			
Age group (months)						
0–11 months	1679	88	229	12	Ref	
12–23 months	1082	87.8	150	12.2	1.0 (0.8–1.3)	
Gender						
Male	1394	50.5	186	49.1	Ref	
Female	1367	49.5	193	50.9	1.1 (0.9–1.3)	
Primary care taker's education						
No education	2281	82.6	315	83.1	Ref	
1–5 years	307	11.1	44	11.6	1 (0.7–1.5)	
6–16 years	173	6.3	20	5.3	0.8 (0.5–1.3)	
Primary wage earner's education						
No education	1454	52.7	217	57.3	Ref	Ref
1–5 years	686	24.9	75	19.8	0.7 (0.6–1)	0.7 (0.6 – 1.0)
6–16 years	621	22.5	87	23.0	0.9 (0.7–1.2)	1 (0.8–1.3)
People in household, median (IQR)	8	6–11	8	6–11	1.0 (1.0–1.0)	
No. of rooms in house	1	1–2	1	1–2	1.0 (0.9–1.1)	
Crowding Index	6	4–7	6	4–7.5	1.0 (1.0–1.1)	
Symptoms (in last 2 weeks) *						
Runny nose	1364	50.6	220	59.3	1.4 (1.1–1.8)	1.5 (1.2–1.9)
Cough	1046	38.8	174	46.9	1.4 (1.1–1.7)	
Fever	1284	47.6	179	48.3	1 (0.8–1.3)	
Fast breathing	65	2.4	14	3.8	1.6 (0.9–2.9)	
Difficulty in breathing	548	20.3	64	17.3	0.8 (0.6–1.1)	0.7 (0.5 – 1.0)
Lower Chest indrawing	61	2.3	13	3.5	1.6 (0.9–2.9)	
Temperature						
Hypothermia	9	0.3	2	0.5	Ref	
Normal temperature	2523	93.6	347	93.5	0.6 (0.1–2.9)	
Hyperthermia	165	6.1	22	5.9	0.6 (0.1–3)	
Tachypnea	182	6.8	31	8.4	1.3 (0.8–1.9)	
Lower chest indrawing	46	1.7	1	0.3	0.2 (0.0–1.1)	
Hospitalization in past 12 months						
No	2678	97.0	368	97.1	Ref	
Yes	83	3.0	11	2.9	1 (0.5–1.8)	
Outpatient visits in past one month						
None	1424	51.6	187	49.3	Ref	
One	703	25.5	94	24.8	1 (0.8–1.3)	
Two or more	634	23.0	98	25.9	1.2 (0.9–1.5)	
Exposure to ETS	993	36.0	123	32.5	0.9 (0.7–1.1)	0.8 (0.6–1)
Cooking Fuel						
Natural gas	431	15.6	54	14.3	Ref	
Wood/paper/straw/crop residue/animal dung	2317	83.9	323	85.2	1.1 (0.8–1.5)	
Other	13	0.5	2	0.5	1.2 (0.3–5.6)	
Child exposed to smoke (<2 m)	1493	54.1	196	51.7	0.9 (0.7–1.1)	
No of PCV 10 doses						
Zero	501	18.2	87	23.0	Ref	Ref
One	289	10.5	61	16.1	1.2 (0.8–1.7)	1.3 (0.9–1.9)
Two	339	12.3	50	13.2	0.8 (0.6–1.2)	1 (0.7–1.4)
Three	1630	59.1	180	47.6	0.6 (0.5–0.8)	0.7 (0.5–1)
Year of enrollment						
2014/15	647	23.4	124	32.7	Ref	Ref
2015/16	678	24.6	102	26.9	0.8 (0.6–1)	0.8 (0.6–1)
2016/17	704	25.5	75	19.8	0.6 (0.4–0.8)	0.6 (0.4–0.8)
2017/18	732	26.5	78	20.6	0.6 (0.4–0.8)	0.6 (0.5–0.9)

*this was not included in multivariable analysis, instead no of PCV doses was used. ETS-Environmental Tobacco Smoke.

Tachypnea is defined as: Children younger than 2 months - Greater than or equal to 60 breaths/min, children aged 2–11 months - Greater than or equal to 50 breaths/min, children aged 12–59 month - Greater than or equal to 40 breaths/min

Hypothermia is defined as underarm temperature below 35.0 °C (95.0 °F), Hyperthermia is defined as underarm temperature ≥38.0 °C (100.4 °F).

## 4 Discussion

Vaccine Type carriage declined significantly in our study population with modest increases in vaccine coverage. When compared to the pre-introduction VT carriage rate of 26.7% in the same population, a greater than 50% decline was observed in the unvaccinated group at third year of the study with a vaccine coverage of 66.0%. This critical cutoff is described in literature as the vaccine threshold required to achieve herd effect. Loughlin et al have reported this threshold to be around 75% in Massachusetts, USA [20]. At the end of 4 years, VT carriage in the unvaccinated declined further to 10.3% - a total of 61.4% decline at 68.4% vaccine coverage.

For all children under 2 years of age, VT carriage was 9.6% at the end of 4 years - a decline of 64% from pre introduction levels. In Sao Paulo, Brazil two years after the introduction of PCV 10, VT carriage declined by more than 90.9% % from 19.8% to 1.8% in children aged 12–23 months. An important difference here is a high vaccine coverage of 93% and a 3 + 1 schedule [21]. In Mongolia which implemented a 2 + 1 regimen for PCV13, a 53.3% % decline in VT carriage from 42.2% to 19.7% was observed one year after introduction in children 12–23 months of age at vaccine coverage of 18% for three doses and 95% for 2 doses. Thus a 2-dose regimen in Mongolia appeared to perform well [22]. In The Gambia VT carriage declined by 65.7%, from 33.3% to 11.4% five years post introduction of PCV13 in the background of more than 95% vaccine coverage and a 3 + 0 schedule [23]. In Lao PDR, which also used PCV13 as a 3 + 0 regimen, a 39.8% decline from 32.9% to 19.8% in VT carriage was observed with greater than 90% vaccine coverage [24]. In Kenya, where they used a 3 + 0 schedule for PCV10, a 74% decline from 33.8% to 8.8% over 7 years with greater than 80% vaccine coverage[25]. Three years after introduction of PCV 10 with a 3 + 0 schedule in Mozambique, vaccine-type carriage declined by almost 43% in both HIV infected and healthy children aged less than 5 years in whom just 34.1% had received three doses of PCV 10 [26]. PCV 10 serotypes declined by 67.2% in Fiji in children aged 1–2 years three years post PCV 10 introduction in a 3 + 0 schedule and vaccine coverage was 100% by the final year of study [27]. As seen above the decline in VT carriage was higher in most of these countries post introduction, that can be most likely attributed to higher vaccine coverage.

To describe the estimates of direct and indirect effect, we took a novel approach using a modified Halloran model which used the pre-introduction carriage levels in the absence of a concurrent unvaccinated population. For the direct effect we compared the vaccinated children (3 doses) against the unvaccinated children (0 dose) in the study population. We looked at indirect effect in two ways- first in the form of decline in the unvaccinated group over the course of four years. Secondly, we calculated a more formal measure of indirect effect by comparing VT carriage in the unvaccinated group (0 dose) with the pre-introduction VT carriage [14]. This is a slightly different approach from other studies reported in literature where the comparison is made usually with the adult population, older children or the group too young to be vaccinated or a concurrent population where the vaccine has not been introduced. These groups would contain vaccinated children overtime and thus underestimate the indirect effect seen in the population. The benefit of including a pre-introduction comparison group of same age is that they would have accrued similar exposure time. Moreover, both the pre and post introduction study samples shared similar baseline characteristics. The vaccine demonstrated significant direct effect which however declined over time due to increasing indirect effect. This is a bit counterintuitive but is in fact a reflection of the herd effect in the unvaccinated group and is in accordance with the Halloran model.

Our estimate of indirect effect was also comparable to other published studies. In Mozambique, three years after the introduction, VT carriage declined by 30% from 39.1% to 27.3% with a vaccine coverage of 34.1%, among HIV-infected unvaccinated children aged 24–59 months [26]. In The Gambia, mothers of fully vaccinated children who received all the PCV doses showed a VT carriage decline of 33.3% from 8.4% to 5.6% [23]. In Kenyan children aged 5–14 years, VT carriage declined by 61.4% from 15.3% to 5.9% and a decline of 75.4% (from 5.7% to 1.4%) was noted in the population aged 15 years and above [25]. In Malawi, VT carriage declined by 63.6% from 21.2% to 7.7% in 5–15 years old children and from 6.6% to 2.4% in mothers of vaccinated children [28]. Similar decline was observed in Fiji in children aged 2–6 years where VT carriage declined by 58% from 21.7% to 9.1% and in their caregivers from 2.4% to 0.8% - a 66.6% decline [27]. In Iceland, children aged 3.5–6.3 years showed a 48.7% decline in VT carriage and in Finland a decline of 29% was reported in older siblings of children who were vaccinated with PCV10 [29], [30]. PCV13 carriage in Norway declined in older than 24-month age population from 14.6% to 4.9% - a decline of 66.4% [31]. A study in Boston reported a sustained 50% reduction in the PCV13 serotype carriage in unvaccinated children at a vaccine coverage of 80% [20]. In Mongolia, where they looked at the indirect effect in children too young to be vaccinated, a 51% decline in VT carriage from 12.9 to 6.3% was observed in children aged 5–8 weeks [22]. In Fiji, the VT carriage declined by 39% from 9.6% to 5.8% in the 5–8 weeks age group [27].

In this study sample, partially vaccinated children (those receiving either one or two doses only) had similar VT carriage rates as the unvaccinated group. This is important in context of reduce dose regimens that have been advocated in many developed countries like UK but might prove to be inefficient in our setting [32].

There was no difference in VT carriage rate when we divided the age into three categories, however looking at the overall pneumococcal carriage, when compared to 4–11 months, the children too young to be vaccinated were less likely to be positive (OR 0.6, 95% CI 0.5–0.8) while children 12–23 months were more likely to be positive (OR 1.3, 95% CI 1.1–1.6). This reflects increased acquisition of *S. pneumoniae* through early infancy and childhood.

The overall carriage in our study was very high and remained high 4 years after introduction which is comparable to what has been reported previously in several other studies from low and middle-income countries. A study in Kenya reported an overall carriage rate of 76% in under 5 children, five years after introduction of PCV10 [25], overall carriage was 85% in The Gambian children aged 6–12 months 5 years after introduction of PCV13 [23], [33]. In Mozambique two years after introduction of PCV10, the overall carriage was 84.8% in children under 5 years of age [26]. This overall carriage is however high in comparison to what has been reported in many of the developed countries. The overall carriage rate in children in Boston two years after PCV 13 introduction was 24%. In the UK, six years after PCV 13 introduction, overall carriage rates in children less than five years were 51.9% whereas in Sweden overall carriage rates were 30% post-PCV introduction [20], [34], [35]. This difference is observed despite high vaccine coverage rates in some of the African populations. Reasons for this are not clearly understood but it might be related to differences in microbial serotype diversity in the regions as well as the pre introduction carriage rates. We saw a modest decline in the overall carriage which was attributable to the decline in vaccine type carriage. Many of the other studies reported either non-significant declines or even increases in overall carriage rates due to increase in serotypes not covered by the vaccine. So as expected, even in our study the decrease in VT carriage was partially offset by increases in NVT and specifically with the three PCV13 serotypes not included in PCV10. Results from serotype distribution show a large proportion contributed by the three PCV13 specific serotypes and nearly 70 percent non-vaccine type serotypes. This points towards a need for development of a vaccine with pan-serotype coverage. The overall carriage decreased over time with no concurrent rise in NVT carriage.

Results from regression modelling show a vaccine effectiveness of 40% for VT carriage, with increasing efficacy with increasing numbers of PCV10 doses. Of the predictors of VT carriage, nothing was associated with carriage except runny nose affirming previous work. Loughlin et al reported having respiratory symptoms at time of enrollment, especially having a runny nose or earache as strong predictors of colonization [20]. Mackenzie et al. showed over 3-fold higher rates of VT carriage in children with runny nose in the previous week [36] and Adetifa et al. showed nearly 50% increased rates of VT carriage in children with recent runny nose [37]. Finn et al reported associations between both rhinitis symptoms and respiratory viral infections and pneumococcal carriage in the nasopharynx. Pneumococcus became more easily detectable in the presence of rhinovirus infection [38]. In our study we found a negative association between ETS and VT carriage which is in contradiction with other reports [39]. Greenberg et al. showed high rates of VT carriage in children exposed to tobacco smoke [40]. We report an inverse relationship which could be spurious in nature or inability of the tool to capture the symptom history accurately.

In analysis for overall carriage, age group 12–23 months and runny nose was positively associated whereas an age group of 0–3 months, higher education status of primary caretaker, difficulty breathing, being enrolled in the third and final years of the study showed a negative association. These results are described in supplement.

This is the first study to come out from Pakistan reporting impact of introduction of PCV10 on nasopharyngeal carriage in children. Riaz et al report impact of PCV10 on Invasive Pneumococcal Disease across a network of hospitals in Karachi. In the case control analysis vaccine effectiveness for three doses was calculated to be 81.9% but with large non-significant confidence intervals because of small sample size [41]. We used standardized WHO and CDC protocols for collection and laboratory analysis. Ours was a large study spread over a period of 4 years which allowed us to look at temporal trends and draw inferences. One limitation of our study was that we did not check for multiple serotypes and carriage density and thus could not look at co-carriage. The serotyping method used allowed for testing of only 39 of the nearly 100 known pneumococcal serotypes. Our study was performed in a small rural district of Matiari which may not be representative of the entire population of Pakistan. Since our study was performed in healthy children, we may have missed to detect highly invasive serotypes like 1, 12F and 5 that commonly cause outbreaks of disease but are rarely carried and may only be transiently detectable in carriage during outbreaks. In cases where vaccination card was not available, vaccine status ascertainment was made solely on the basis of recall and common practices which may have resulted in misclassification though we tried to address this by performing various sensitivity analysis. In our study we report a high prevalence of respiratory symptoms at the time of enrolment which could be due to over reporting by the caregivers.

We acknowledge that vaccine coverage might have declined after the study was completed and there is a need for continuous surveillance to detect change in carriage distribution.

## 5 Conclusion

Our study provides evidence that a 3 + 0 schedule works well in the setting of a low middle income country in providing both direct and indirect protection to children. To maximally capitalize on these benefits, a high and sustained vaccine coverage with continuous monitoring for disease and carriage prevalence for emerging serotypes is required.

## Funding

This work was supported by Bill & Melinda Gates Foundation through grant ID #OPP1111303.

## CRediT authorship contribution statement

**Muhammad Imran Nisar:** Conceptualization, Methodology, Formal analysis, Writing - original draft, Writing - review & editing, Visualization, Supervision, Project administration, Funding acquisition. **Sheraz Ahmed:** Writing - review & editing, Supervision, Project administration. **Fyezah Jehan:** Conceptualization, Methodology, Writing - review & editing, Funding acquisition. **Shahira Shahid:** Writing - original draft, Writing - review & editing, Visualization. **Sadia Shakoor:** Methodology, Writing - review & editing, Investigation. **Furqan Kabir:** Writing - review & editing, Investigation, Resources, Supervision. **Aneeta Hotwani:** Writing - review & editing, Investigation, Resources. **Sahrish Munir:** Writing - review & editing, Investigation, Resources. **Sajid Muhammad:** Writing - review & editing, Data curation, Software, Formal analysis, Visualization. **Farah Khalid:** Writing - review & editing, Data curation, Software, Formal analysis, Visualization. **Benjamin Althouse:** Writing - review & editing, Formal analysis, Visualization. **Hao Hu:** Writing - review & editing, Formal analysis, Visualization. **Cynthia Whitney:** Writing - review & editing, Methodology, Resources, Supervision. **Asad Ali:** Writing - review & editing, Conceptualization, Methodology, Funding acquisition, Supervision. **Anita K.M. Zaidi:** Writing - review & editing, Supervision. **Saad B. Omer:** Writing - review & editing, Supervision. **Najeeha Iqbal:** Writing - review & editing, Methodology, Formal analysis, Supervision.

## Declaration of Competing Interest

The authors declare that they have no known competing financial interests or personal relationships that could have appeared to influence the work reported in this paper.
