# Supplementary material for: Direct and indirect effect of 10 valent pneumococcal vaccine on nasopharyngeal carriage in children under 2 years of age in Matiari, Pakistan
Source: Vaccine. 2021 Feb 22;39(8):1319–27. doi: 10.1016/j.vaccine.2020.12.066 (PMC7910277; doi:10.1016/j.vaccine.2020.12.066)
Supplement: Supplementary data 2 [file mmc2.doc]

**Figure s1-Map of study site**


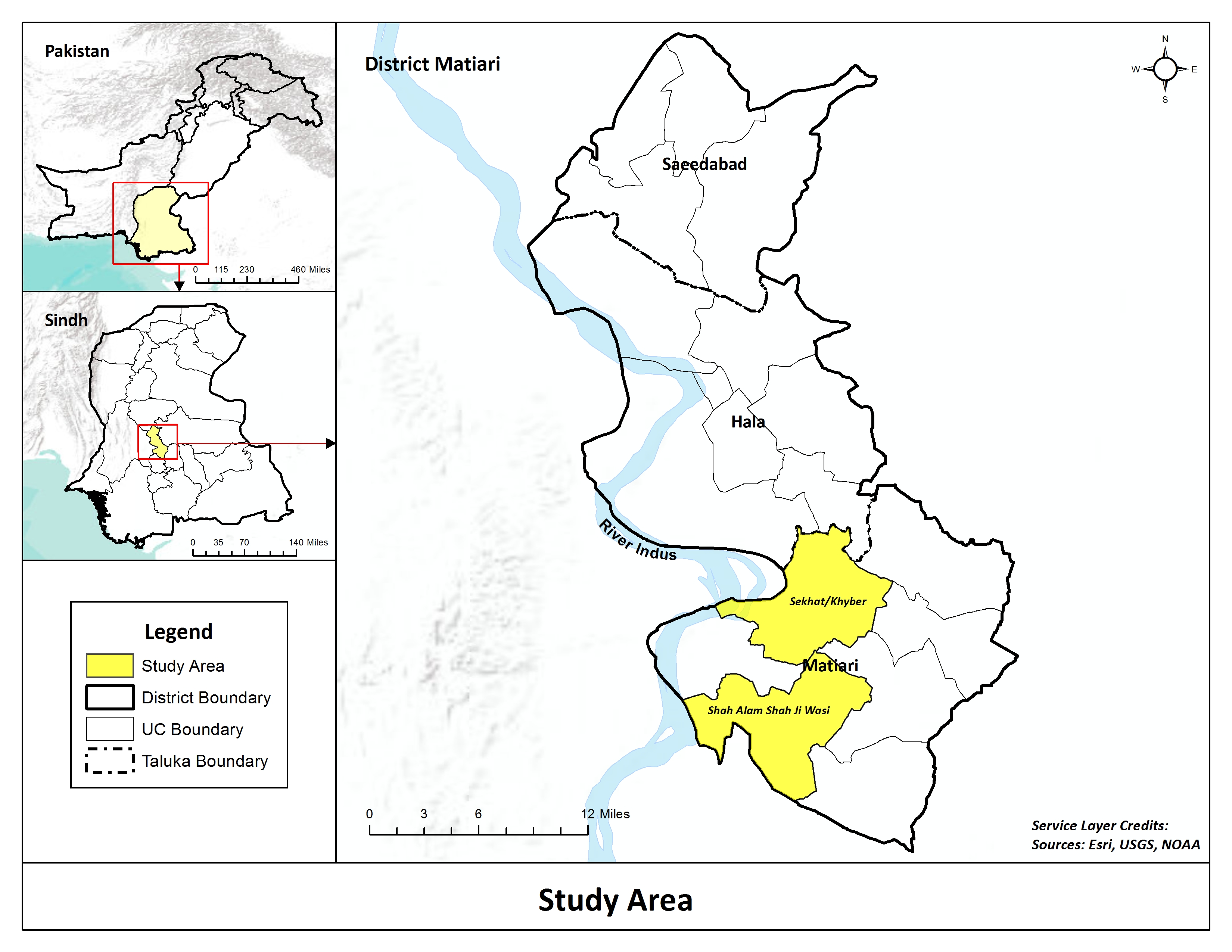


**Figure s2- Percent of children carrying any serotype, vaccine type serotype and non-vaccine type serotype by year of the study.**


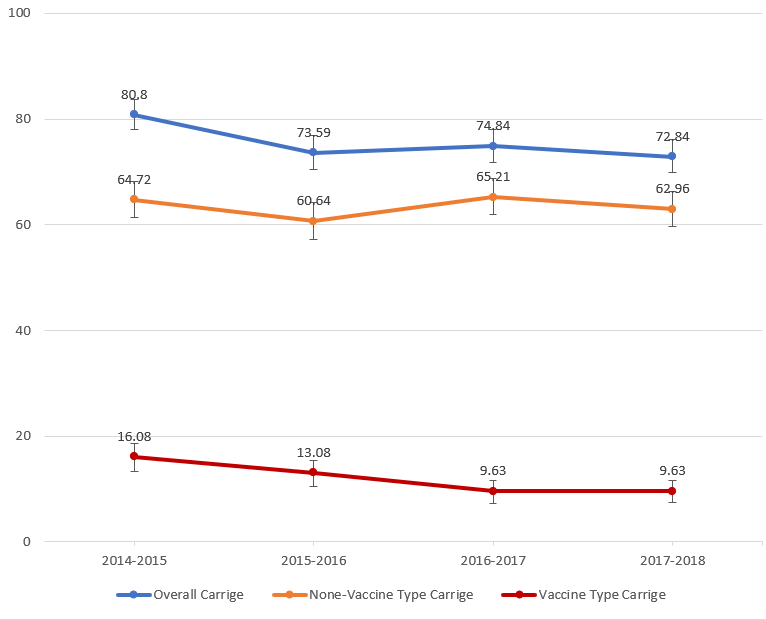


**Figure s3 – Serotype distribution over the years (2014-2018)**


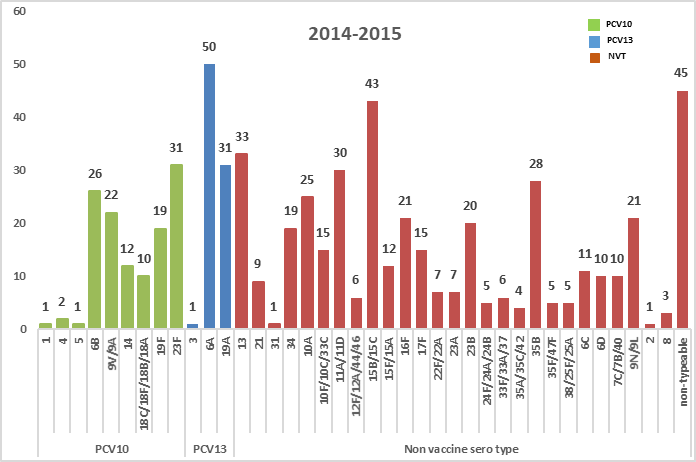

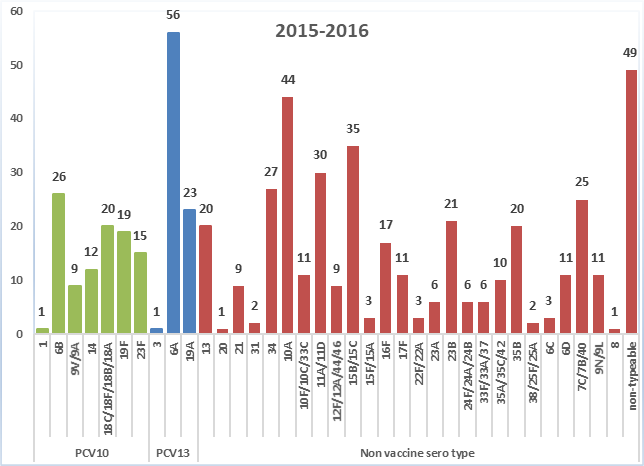


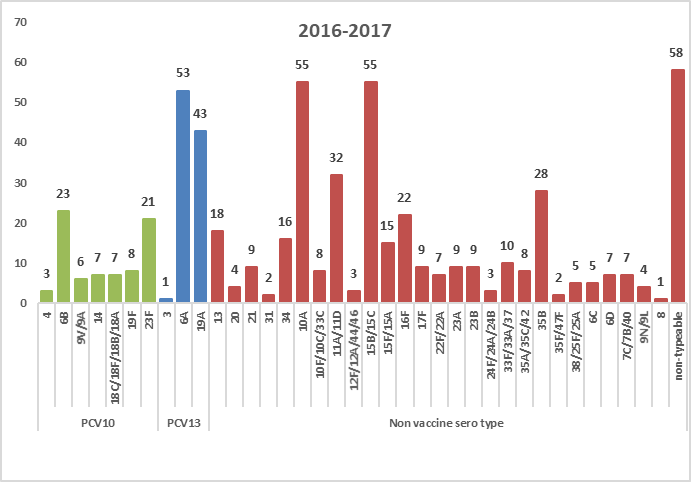

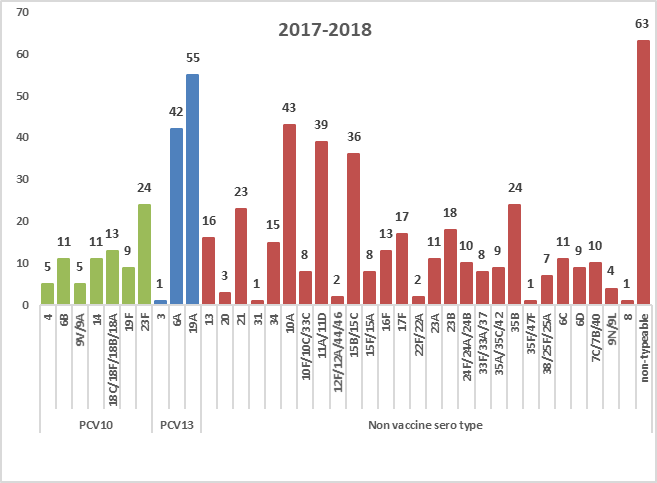


**Figure S4-Age distribution of PCV10 doses (card-verified)- red, green and blue vertical lines denote 6, 10 and 14 week where the doses should ideally be received**

**Table S1- Age distribution of PCV10 doses (card-verified)**

|  | **N** | **Min** | **Max** | **Median (IQR)** | **Number of PCV doses according to Pakistan’s EPI (Weeks)** |
| --- | --- | --- | --- | --- | --- |
| **Dose 1** | 2022 | 5.0 | 81.9 | 9.4 (7.4,13.0) | 6 |
| **Dose 2** | 1776 | 9.0 | 87.1 | 15.1 (12.6,19.9) | 10 |
| **Dose 3** | 1480 | 13.0 | 91.6 | 20.9 (17.6,26.5) | 14 |

**Table S2- Number of vaccine doses received among study participants by year**

| **No. of PCV doses** |  | **2014-2015 (n=771)** | **2015-2016 (n=780)** | **2016-2017 (n=779)** | **2017-2018 (n=810)** | **2014-2018 (n=3140)** |
| --- | --- | --- | --- | --- | --- | --- |
| **0 dose %**  **(95% CI)** | (verbal/card verified) | 35.0  (31.7-38.4) | 19.9  (17.1-22.8) | 11.0  (8.9-13.5) | 9.5  (7.6-11.7) | 18.7  (17.4-20.1) |
| **1 dose %**  **(95% CI)** | (verbal/card verified) | 11.0  (8.9-13.4) | 13.8  (11.5-16.5) | 10.9  (8.8-13.3) | 9.0  (7.1-11.2) | 11.2  (10.1-12.3) |
| **2 doses %**  **(95% CI)** | (verbal/card verified) | 13.0  (10.7-15.5) | 11.7  (9.5-14.1) | 12.1  (9.9-14.6) | 13.1  (10.8-15.6) | 12.5  (11.3-13.7) |
| **3 doses %**  **(95% CI)** | (verbal/card verified) | 41.0  (37.5-44.6) | 54.6  (51.0-58.2) | 66.0  (62.5-69.3) | 68.4  (65.1-71.6) | 57.6  (55.9-59.4) |
| **1 dose %**  **(95% CI)** | (card verified) | 7.5  (5.8-9.6) | 11.3  (9.1-13.7) | 9.4  (7.4-11.6) | 7.5  (5.8-9.6) | 8.9  (7.9-10.0) |
| **2 doses %**  **(95% CI)** | (card verified) | 8.2  (6.3-10.3) | 8.8  (6.9-11.1) | 10.9  (8.8-13.3) | 12.2  (10.0-14.7) | 10.1  (9.0-11.2) |
| **3 doses %**  **(95% CI)** | (card verified) | 28.8  (25.6-32.1) | 45.0  (41.5-48.6) | 58.2  (54.6-61.6) | 57.9  (54.4-61.3) | 47.6  (45.9-49.4) |

p-value for trend 0.001

**Figure S5- VT carriage by age in months**


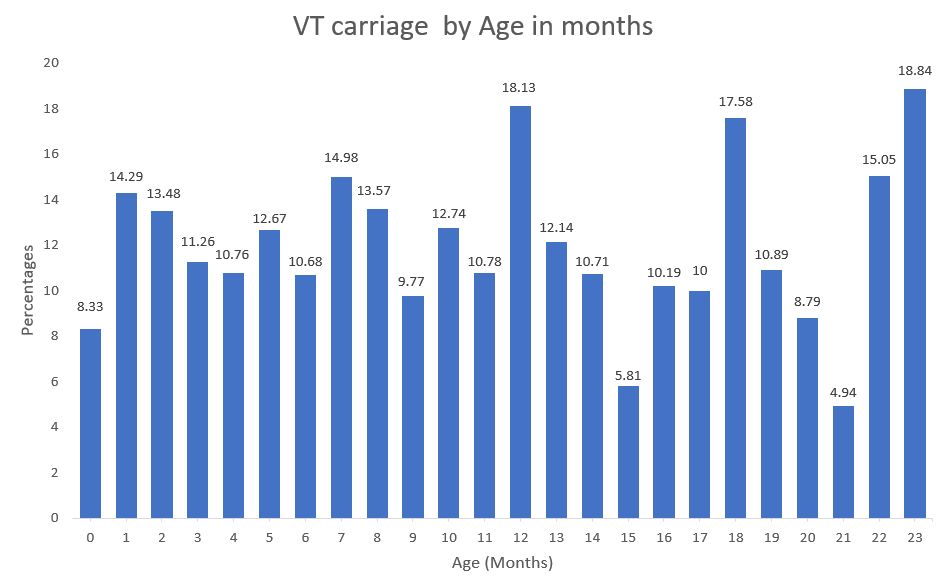


Figure s6- Sample collection and storage


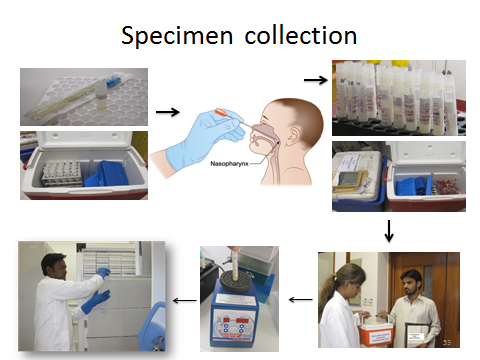


**Figure s7- sample processing and serotyping**


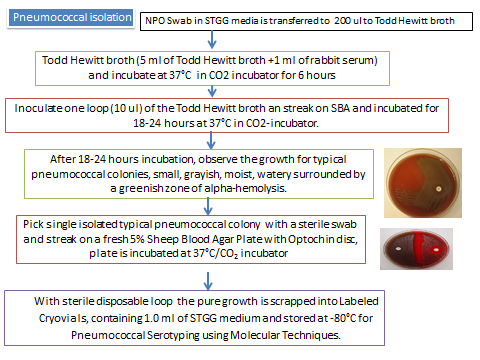


**Figure s8– sequential multiplex PCR**


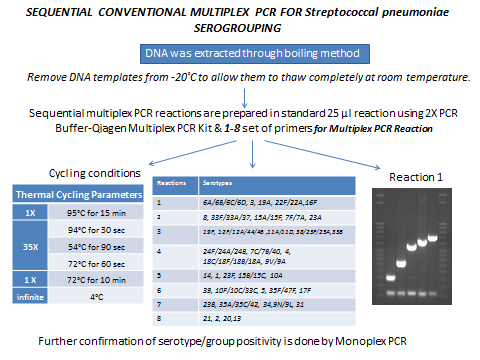


**Table s3- VT carriage based on number of PCV10 doses**

| **Year** |  | **2014-2015** | **2015-2016** | **2016-2017** | **2017-2018** | **2014-2018** |
| --- | --- | --- | --- | --- | --- | --- |
| **VT carriage rate (0 dose) %** | (card-verified) | 16.6 (13.2-20.5) | 16.2 (12.0-21.1) | 10.1 (6.0-15.7) | 11.0 (6.9-16.5) | 14.5 (12.4-16.8) |
| **VT carriage rate (1 dose) %** | (card-verified) | 24.1 (13.9-37.2) | 9.1 (4.0-17.1) | 15.1 (7.8-25.4) | 14.8 (7.0-26.2) | 15.0 (11.0-19.7) |
| **VT carriage rate (2 doses) %** | (card-verified) | 20.6 (11.5-32.7) | 8.7 (3.3-18) | 8.2 (3.4-16.2) | 11.1 (5.7-19) | 11.7 (8.4-15.8) |
| **VT carriage rate (3 doses) %** | (card-verified) | 11.7 (7.8-16.7) | 12.5 (9.3-16.5) | 8.8 (6.4-11.8) | 8.1 (5.8-11.0) | 9.9 (8.4-11.5) |
| **VT carriage rate (1 dose) %** | (verbal/card-verified) | 24.7 (16.0-35.3) | 14.8 (8.7-22.9) | 14.1 (7.5-23.4) | 16.4 (8.8-27) | 17.4 (13.6-21.8) |
| **VT carriage rate (2 doses) %** | (verbal/card-verified) | 20.0 (12.7-29.2) | 12.1 (6.2-20.6) | 7.4 (3.0-14.7) | 12.3 (6.7-20.1) | 13.0 (9.9-16.8) |
| **VT carriage rate (3 doses) %** | (verbal/card-verified) | 11.4 (8.1-15.4) | 12.4 (9.5-16.0) | 8.9 (6.6-11.8) | 8.1 (6-10.7) | 9.9 (8.6-11.4) |

**Table s4- Direct, Indirect, Total and Overall effect of PCV10 on VT carriage (0 dose vs 1,2 or 3 doses)**

| **Year** | **2014-2015** | **2015-2016** | **2016-2017** | **2017-2018** | **2014-2018** |
| --- | --- | --- | --- | --- | --- |
| **Direct Effect (95% CI)** | 11.7  (-22.9- 36.6) | 9.8  (-39.7-41.7) | 19.3  (-50.9-56.9) | 8.0  (-83.7 - 54.0) | 22.6  (3.4- 38.0) |
| **Indirect effect (95% CI)** | 34.7  (3.3-45.5) | 46.7  (17.0- 65.8) | 56.3  (18.8-76.5) | 61.0  (22.2-80.4) | 44.5  (25.7-58.5) |
| **Total effect (95% CI)** | 42.3  (22.3-57.2) | 52  (35.3-64.3) | 64.8  (51.7-74.3) | 64.1  (51.1-73.7) | 57.0  (45.3-66.3) |
| **Overall effect (95% CI)** | 39.7  (21.0- 54.0) | 50.8  (34.8-62.9) | 65.1  (52.4-74.3) | 63.9  (51.1-73.4) | 54.9  (42.9-64.4) |

Direct effect was calculated as 1– (VT carriage rate in children who received 1,2 or 3 doses /VT carriage rate in children who received zero dose), indirect effect was defined as 1- (VT carriage rate in in children who received zero dose /26.7%), total effect was defined as 1-(VT carriage rate in children who received all 1,2 or 3 /26.7%) and overall effect was defined as 1- ( carriage rate in the study population / 26.7%).

**Table s5 Direct, Indirect, Total and Overall effect of PCV10 on VT carriage (0 dose vs 3 doses) Age restricted (>=4 months)**

| **Year** | **2014-2015** | **2015-2016** | **2016-2017** | **2017-2018** | **2014-2018** |
| --- | --- | --- | --- | --- | --- |
| **N** | 716 | 666 | 719 | 735 | 2613 |
| **VT carriage in 3 doses %^α^ (95% CI)** | 11.4 (8.1-15.4) | 12.4 (9.5-16.0) | 9.0 (6.7-11.8) | 7.9 (5.7-10.4) | 9.9 (8.6-11.4) |
| **VT carriage in 2 doses % ^∞^ (95% CI)** | 17.8 (10.5-27.3) | 12.0 (5.9-21.0) | 8.4 (3.5-16.6) | 14.1 (7.5-23.4) | 13.2 (9.8-17.3) |
| **VT carriage in 1 dose %* (95% CI)** | 24.2 (14.5-36.4) | 17.8 (9.8-28.5) | 17.5 (8.7-29.9) | 17.8 (8.0-32.1) | 19.5 (14.7-25.1) |
| **VT carriage in 0 dose %^β^ (95% CI)** | 18.4 (13.8-23.9) | 15.5 (8.5-25.0) | 13.0 (6.1-23.3) | 10.3 (3.9-21.2) | 16.0 (12.8-19.7) |
| **Direct Effect (95% CI)** | 38.2 (7.4-58.8) | 19.6 (-40.7-54.1) | 30.8 (-35.0-64.6) | 24.0 (-70.8-66.2) | 38.3 (20.6-52.1) |
| **Indirect effect (95% CI)** | 30.9 (25.1-36.7) | 42.0 (31.5-52.6) | 51.1 (39.4-62.9) | 61.3 (48.7-73.8) | 39.9 (35.4-44.4) |
| **Total effect (95% CI)** | 57.3 (51.9-62.8) | 53.4 (48.7-58.1) | 66.2 (62.1-70.3) | 70.6 (66.7-74.4) | 62.9 (60.7-65.2) |
| **Overall effect (95% CI)** | 40.9 (37.3-44.5) | 50.0 (46.2-53.8) | 62.5 (59.0-66.0) | 64.8 (61.4-68.3) | 54.7 (52.9-56.6) |

**^α^** p-value for trend 0.021,  **^∞^**p-value for trend 0.357, *p-value for trend 0.379 , **^β^** p-value for trend 0.088

**Table s6 Direct, Indirect, Total and Overall effect of PCV10 on VT carriage (1,2 and 3 doses vs 0 dose) (Age restricted >=4 months)**

| **Year** | **2014-2015** | **2015-2016** | **2016-2017** | **2017-2018** | **2014-2018** |
| --- | --- | --- | --- | --- | --- |
| **N** | 716 | 666 | 719 | 735 | 2613 |
| **Direct Effect (95% CI)** | 21.9(-10.1-44.6) | 15.6 (-45.1-50.9) | 25.7 (-42.7-61.3) | 10.0 (-98.9-59.3) | 29.3 (10.3-44.3) |
| **Indirect effect (95% CI)** | 30.9 (25.1-36.7) | 42.0 (31.5-52.6) | 51.1 (39.4-62.9) | 61.3 (48.7-73.8) | 39.9 (35.4-44.4) |
| **Total effect (95% CI)** | 46.0 (41.5-50.5) | 51.1 (47.0-55.2) | 63.7 (60.0-67.4) | 65.1 (61.6-68.7) | 57.5 (55.5-59.5) |
| **Overall effect (95% CI)** | 40.9 (37.3-44.5) | 50 (46.2-53.8) | 62.5 (59.0-66.0) | 64.8 (61.4-68.3) | 54.7 (52.9-56.6) |

**Table S7- Predictors of Overall carriage in children less than 2 years of age, n= 3140**

|  | **Overall PCV serotype carriage** | | | | **Unadjusted** | **Adjusted Model** |
| --- | --- | --- | --- | --- | --- | --- |
|  | **Negative** | | **Positive** | |  |  |
|  | **N** | **(%)** | **N** | **(%)** | **OR (95% CI)** | **OR (95% CI)** |
| **N** | 770 |  | 2370 |  |  |  |
| **Age group(months)** |  |  |  |  |  |  |
| **0-11 months** | 519 | 27.2 | 1,389 | 72.8 | Ref | Ref |
| **12-23 months** | 251 | 20.4 | 981 | 79.6 | 1.5 (1.2 - 1.7) | 1.4 (1.1 - 1.6) |
| **Gender** |  |  |  |  |  |  |
| **Male** | 375 | 48.7 | 1,205 | 50.8 | Ref |  |
| **Female** | 395 | 51.3 | 1,165 | 49.2 | 0.9 (0.8 - 1.1) |  |
| **Primary care taker's education** |  |  |  |  |  |  |
| **no education** | 611 | 79.4 | 1,985 | 83.8 | Ref | Ref |
| **1-5 years** | 94 | 12.2 | 257 | 10.8 | 0.8 (0.7 - 1.1) | 0.9 (0.7 - 1.2) |
| **6-16 years** | 65 | 8.4 | 128 | 5.4 | 0.6 (0.4 - 0.8) | 0.6 (0.4 - 0.8) |
| **Primary wage earner's education** |  |  |  |  |  |  |
| **no education** | 383 | 49.7 | 1,288 | 54.3 | Ref |  |
| **1-5 years** | 200 | 26.0 | 561 | 23.7 | 0.8 (0.7 - 1) |  |
| **6-16 years** | 187 | 24.3 | 521 | 22.0 | 0.8 (0.7 - 1) |  |
|  |  |  |  |  |  |  |
| **Total people in household, median (IQR)** | 8 | 6-11 | 8 | 6-11 | 1.0 (0.9 - 1) |  |
| **No. of rooms in house** | 1 | 1-2 | 1 | 1-2 | 0.9 (0.86 - 0.99) |  |
| **Crowding Index** | 5 | 4-7 | 5.5 | 4-7 | 1 (1 - 1) |  |
| **Symptoms (in last 2 weeks) *** |  |  |  |  |  |  |
| **Runny nose** | 329 | 44.1 | 1,255 | 54.0 | 1.5 (1.3 - 1.8) | 1.7 (1.4 - 2) |
| **Cough** | 264 | 35.4 | 956 | 41.2 | 1.3 (1.1 - 1.5) |  |
| **Fever** | 367 | 49.2 | 1,096 | 47.2 | 0.9 (0.8 - 1.1) |  |
| **Fast breathing** | 20 | 2.7 | 59 | 2.5 | 0.9 (0.6 - 1.6) |  |
| **Difficulty in breathing** | 169 | 22.7 | 443 | 19.1 | 0.8 (0.7 - 1) | 0.7 (0.5 - 0.8) |
| **Lower Chest indrawing** | 23 | 3.1 | 51 | 2.2 | 0.7 (0.4 - 1.2) |  |
| **Temperature** |  |  |  |  |  |  |
| **Hypothermia** | 2 | 0.3 | 9 | 0.4 | Ref |  |
| **Normal temperature** | 684 | 91.7 | 2,186 | 94.1 | 0.7 (0.2 - 3.3) |  |
| **Hyperthermia** | 60 | 8.0 | 127 | 5.5 | 0.5 (0.1 - 2.2) |  |
| **Tachypnea** | 45 | 6.0 | 168 | 7.2 | 1.2 (0.9 - 1.7) |  |
| **Lower chest indrawing** | 16 | 2.1 | 31 | 1.3 | 0.6 (0.3 - 1.1) |  |
| **Hospitalization in past 12 months or since birth** |  |  |  |  |  |  |
| **No** | 745 | 96.8 | 2,301 | 97.1 | Ref |  |
| **Yes** | 25 | 3.2 | 69 | 2.9 | 0.9 (0.6 - 1.4) |  |
| **Outpatient visits in past one month** |  |  |  |  |  |  |
| **Zero visit** | 369 | 47.9 | 1,242 | 52.4 | Ref |  |
| **one visit** | 207 | 26.9 | 590 | 24.9 | 0.8 (0.7 - 1) |  |
| **two or more** | 194 | 25.2 | 538 | 22.7 | 0.8 (0.7 - 1) |  |
| **Exposure to Environmental Tobacco Smoke** | 293 | 38.1 | 823 | 34.7 | 0.9 (0.7 - 1) |  |
| **Cooking Fuel** |  |  |  |  |  |  |
| **Natural gas** | 126 | 16.4 | 359 | 15.1 | Ref |  |
| **Wood/paper/straw/crop residue/animal dung** | 637 | 82.7 | 2,003 | 84.5 | 1.1 (0.9 - 1.4) |  |
| **Other** | 7 | 0.9 | 8 | 0.3 | 0.4 (0.1 - 1.1) |  |
| **Child exposed to smoke (less than 2 m)** | 396 | 51.4 | 1,293 | 54.6 | 1.1 (1 - 1.3) |  |
| **Vaccinated** | 436 | 56.6 | 1,374 | 58.0 | 1.1 (0.9 - 1.2) |  |
| **No of PCV 10 doses** |  |  |  |  |  |  |
| **Zero** | 150 | 19.5 | 438 | 18.5 | Ref |  |
| **One** | 91 | 11.8 | 260 | 11.0 | 1 (0.7 - 1.3) |  |
| **Two** | 93 | 12.1 | 298 | 12.6 | 1.1 (0.8 - 1.5) |  |
| **Three** | 436 | 56.6 | 1,374 | 58.0 | 1.1 (0.9 - 1.3) |  |
| **Time** |  |  |  |  |  |  |
| **Year 1** | 148 | 19.2 | 623 | 26.3 | Ref | Ref |
| **Year 2** | 206 | 26.8 | 574 | 24.2 | 0.7 (0.5 - 0.8) | 0.6 (0.5 - 0.8) |
| **Year 3** | 196 | 25.5 | 583 | 24.6 | 0.7 (0.6 - 0.9) | 0.7 (0.6 - 1) |
| **Year 4** | 220 | 28.6 | 590 | 24.9 | 0.6 (0.5 - 0.8) | 0.7 (0.5 - 0.9) |

*this was not included in multivariable analysis, instead no of PCV doses was used. ETS-Environmental Tobacco Smoke

Tachypnea is defined as: Children younger than 2 months - Greater than or equal to 60 breaths/min, children aged 2-11 months - Greater than or equal to 50 breaths/min, children aged 12-59 month - Greater than or equal to 40 breaths/min

Hypothermia is defined as underarm temperature below 35.0° C (95.0 °F), Hyperthermia is defined as underarm temperature ≥ 38.0° C (100.4° F)

Formulas used

**Direct effect**

Direct effect was calculated as 1– (VT carriage rate in children who received all three doses /VT carriage rate in children who received zero dose) or

Direct effect was calculated as 1– (VT carriage rate in children who received 1,2, or 3 doses /VT carriage rate in children who received zero dose).

**Indirect effect**

Indirect effect was defined as 1- (VT carriage rate in in children who received zero dose /26.7%)

**Total effect**

Total effect was defined as 1-(VT carriage rate in children who received all three doses/26.7%) or

Total effect was defined as 1-(VT carriage rate in children who received 1, 2 or 3 doses/26.7%)

**Overall effect**

Overall effect was defined as 1- ( carriage rate in the study population / 26.7%).
